# Evolution of peripheral nerve changes in early multiple sclerosis—a longitudinal MR neurography study

**DOI:** 10.3389/fneur.2024.1335408

**Published:** 2024-05-03

**Authors:** Olivia Foesleitner, Jennifer C. Hayes, Markus Weiler, Georges Sam, Brigitte Wildemann, Wolfgang Wick, Martin Bendszus, Sabine Heiland, Laura Bettina Jäger

**Affiliations:** ^1^Department of Neuroradiology, Heidelberg University Hospital, Heidelberg, Germany; ^2^Department of Neurology, Heidelberg University Hospital, Heidelberg, Germany; ^3^Clinical Cooperation Unit Neuro-Oncology, German Cancer Consortium (DKTK), German Cancer Research Center (DKFZ), Heidelberg, Germany

**Keywords:** magnetic resonance imaging, MR neurography, multiple sclerosis, peripheral nervous system, T2 relaxometry

## Abstract

**Objectives:**

Multiple sclerosis (MS) is a demyelinating disorder of the central nervous system. Increasing evidence indicates additional peripheral nerve involvement in early and chronic disease stages. To investigate the evolution of peripheral nerve changes in patients first diagnosed with MS using quantitative MR neurography.

**Materials and methods:**

This prospective study included 19 patients with newly diagnosed MS according to the revised McDonald criteria (16 female, mean 30.2 ± 7.1 years) and 19 age-/sex-matched healthy volunteers. High-resolution 3 T MR neurography of the sciatic nerve using a quantitative T2-relaxometry sequence was performed, which yielded the biomarkers of T2 relaxation time (T2app) and proton spin density (PSD). Follow-up scans of patients were performed after median of 12 months (range 7–16). Correlation analyses considered clinical symptoms, intrathecal immunoglobulin synthesis, nerve conduction study, and lesion load on brain and spine MRI.

**Results:**

Patients showed increased T2app and decreased PSD compared to healthy controls at initial diagnosis and follow-up (*p* < 0.001 each). Compared to the initial scan, T2app further increased in patients at follow-up (*p* = 0.003). PSD further declined by at least 10% in 9/19 patients and remained stable in another 9/19 patients. Correlation analyses did not yield significant results.

**Conclusion:**

Peripheral nerve involvement in MS appears at initial diagnosis and continues to evolve within 1 year follow-up with individual dynamics. Quantitative MRN provides non-invasive biomarkers to detect and monitor peripheral nerve changes in MS.

## Highlights

- Growing evidence documents peripheral nerve involvement in multiple sclerosis (MS) already at early disease stages, however, with so far unknown longitudinal development.
- This first longitudinal MR neurography study in multiple sclerosis reveals evolution of quantitative peripheral nerve changes within 1 year follow-up.
- Quantitative MRN provides novel non-invasive biomarkers to detect and monitor peripheral nerve involvement in MS.

## Introduction

Multiple sclerosis (MS) is a demyelinating disease of the central nervous system (CNS). Evidence of spatial and temporal distribution of inflammatory CNS lesions is required according to the revised McDonald criteria of 2017 (1). Recently, there is growing evidence indicating an additional involvement of the peripheral nervous system (PNS) in early and chronic MS, as shown by recent MR studies (2–4), electrophysiology (5–8) as well as neuropathology (9, 10). Understanding the etiology and pathophysiology of these findings is relevant to prevent potentially irreversible peripheral nerve affection.

High-resolution MR neurography (MRN) is a non-invasive imaging method to detect pathological changes in peripheral nerves at high diagnostic sensitivity (11–13). In addition to conventional fat-saturated T2-weighted sequences, more recent quantitative MRN offers the potential to provide novel biomarkers of nerve tissue integrity. As such, quantitative T2-relaxometry sequences have been successfully applied in peripheral neuropathies of various etiologies (14–18). Based on the density and macromolecular composition, the water content of nerve tissue can be estimated, and thus specific patterns of structural nerve damage identified. Thereby, predominant demyelination as opposed to axonal loss can be differentiated.

MRN studies showed peripheral nerve changes in patients with chronic MS but also already at initial diagnosis (2–4). These findings favor a primary inflammatory co-affection of the PNS over secondary Wallerian degeneration or medication-induced effects. However, so far evidence is based on cross-sectional studies. Hence, the aims of the present longitudinal study are to investigate the evolution of peripheral nerve changes in MS patients at initial diagnosis and at 12 months follow-up in correlation with clinical/neurological features, laboratory parameters as well as lesion load on brain and spine MRI.

## Materials and methods

### Study design and participants

This prospective, longitudinal, observational study was performed at a single academic medical center following the STROBE guidelines (19). A consecutive sample of 19 patients with MS according to the revised McDonald criteria of 2017 (1) were enrolled at their first clinical presentation during their in-patient stay from 10/2020-08/2022 (flow chart provided in the Supplementary material). Exclusion criteria were any concurrent differential diagnosis to MS, risk factors for peripheral neuropathy such as vitamin deficiency and endocrinological disorders, prior medication with potentially neurotoxic effects, and general MRI contraindications. In addition, 19 demographically matched healthy volunteers without any history of neurological diseases were recruited by public announcement. The study was approved by the local ethics committee (S-405/2012, updated 2018 and 2023) and performed in accordance with the Declaration of Helsinki 2013. Written informed consent was obtained from all participants.

### Clinical examinations

Detailed medical history and comprehensive neurological examination were documented for all patients at initial presentation. Cerebrospinal fluid (CSF) cell count was determined using a Fuchs-Rosenthal chamber (upper limit of normal: 4 cells/μL) and CSF cytology was performed after May–Grünwald staining of individual cytospins. Concentrations of immunoglobulin (Ig) G and IgM were measured in serum and in CSF by standard nephelometric assays to calculate the CSF-to-serum quotients. Intrathecal IgG and IgM synthesis was quantified with the formula by Reiber (20). If quantitative intrathecal IgG synthesis yielded negative results, presence of oligoclonal bands was assessed with isoelectric focusing followed by immunoblotting and considered positive in cases of patterns 2 or 3 (21). Visual evoked potential analysis for P100 latency and amplitude was performed as part of clinical routine in 16/19 patients. Extended nerve conduction studies (NCS) of the same leg as examined with MRN were performed in 18/19 patients. The NCS protocol included distal motor latencies, compound muscle action potentials, and F-waves of the tibial and peroneal nerves, nerve conduction velocities of the tibial, peroneal, and sural nerves, as well as sensory nerve action potentials of the sural nerve. In all NCS, skin temperature was controlled at a minimum of 32°C. The administered dose of intravenous corticosteroids during stationary therapy at initial diagnosis was documented for all patients. At follow-up, patients were asked to complete a questionnaire about any residual or new clinical symptoms and about previous disease-modifying therapies.

### CNS imaging

Contrast-enhanced brain MRI including high-resolution T1-, T2-weighted and fluid-attenuated inversion recovery (FLAIR) sequences as well as spine MRI studies with sagittal and axial T1- and T2-weighted sequences as available were used to determine the CNS lesion load at initial presentation and at follow-up, i.e., the number of T2- and contrast-enhancing T1-lesions.

### MRN acquisition

High-resolution MRN of patients and healthy volunteers was performed in supine position at a 3 Tesla scanner (MAGNETOM Prisma, Siemens Healthineers, Erlangen, Germany) using a 15-channel transmit/receive array knee coil (Siemens Healthineers). For standardization, the left distal thigh at comparable height was examined in all subjects unless patients had symptoms in only the right lower extremity (clinical data is outlined in Table 1). An axial dual-echo turbo-spin-echo 2D sequence was acquired with repetition time: 5,860 ms, echo times: TE_1_ = 14 ms and TE_2_ = 86 ms, field-of-view: 140 × 140 mm^2^, matrix size: 512 × 512, slice thickness: 3.5 mm, interslice gap: 0.385 mm, flip angle: 180°, spectral fat saturation, and an acquisition time of 8:06 min. At follow-up, patients were examined using identical sequence parameters as in their first scan.

**Table 1:** Individual clinical characteristics of patients.

Interne ID	ID	Age/Sex	Diagnosis	At initial diagnosis													At follow-up					
				Neurological examination	Symptom duration (days)	EDSS	CSF (IgG)	CSF (IgM)	Nerve conduction study	Visual evoked potentials	Brain MRI T2w-lesions	Brain MRI CE+ lesions	Spine MRI T2w-lesions	Spine MRI CE+ lesions	McDonald criteria (2017)	Corticosteroids prior to first MRN (g)	FU (months)	Symptoms at FU	Brain MRI	Spine MRI	DMT	Corticosteroid therapy after initial diagnosis
12	1	35/M	MS	Left optic neuritis	7	2.0	OCB+ M3	Normal	Normal	Abnormal (bilateral)	23	Yes	—	—	DIS (MRI), DIT (MRI, CSF)	3.0	16	None	Several new T2-lesions (9 m after FU)	2 new T2-lesions (9 m after FU)	Dimethyl fumarate for 12 m	—
15	2	36/F	MS	Right optic neuritis	5	2.0	Intrathecal IgG synthesis (63.23%)	Normal	Normal	Abnormal (bilateral)	8	Yes	—	—	DIS (MRI), DIT (MRI, CSF)	5.0	16	None	No new lesions (same day as FU)	—	Teriflunomide for 14 m	—
18	3	25/F	MS	Right optic neuritis	7	3.0	Intrathecal IgG synthesis (41.15%)	Normal	Absence of F waves in left peroneal nerve	Abnormal (right)	33	Yes	—	—	DIS (MRI), DIT (MRI, CSF)	4.0	12	None	—	—	Dimethyl fumarate	—
21	4	37/F	MS	Left optic neuritis	7	3.0	Intrathecal IgG synthesis (12.36%)	Normal	Normal	Abnormal (bilateral)	35	No	0	No	DIS (MRI), DIT (CSF)	1.0	14	None	No new lesions (5 m prior to FU)	—	Dimethyl fumarate for 15 m	—
23	5	32/F	MS	Left sixth nerve palsy, subclinical bilateral optic neuritis	9	2.0	Intrathecal IgG synthesis (22.58%)	Normal	A-waves in left peroneal nerve	Abnormal (bilateral)	4	No	3	Yes	DIS (MRI), DIT (MRI, CSF)	2.5	15	None	Two new T2-lesions, CE-(4 m after FU)	no new lesions (4 m after FU)	Teriflunomide for 7 m	—
26	6	42/F	MS	Left hemiparesis (MRC 4-/5), hypoesthesia in right leg, diplopia in left gaze	30	3.0	OCB+ M2	Normal	Normal	Normal	17	No	0	No	DIS (MRI), DIT (CSF)	1.0	12	Persistent hypoesthesia in right leg	No new lesions (11 m after FU)	No new lesions (11 m after FU)	Teriflunomide for 12 m	—
28	7	32/F	MS	Bilateral hyperalgesia below thoracic level 12	6	3.0	OCB+ M2	Normal	Normal	Abnormal (bilateral)	32	No	2	Yes	DIS (MRI), DIT (MRI, CSF)	1.0	12	Neuropathic pain in both legs	No new lesions (5 m after FU)	No new lesions (5 m after FU)	Teriflunomide for 6 m, Diroximel fumerate	Corticosteroid therapy 6 m and 7 m prior to FU
32	8	20/F	MS	Vertigo, gait disturbance	21	2.0	OCB+ M2, intrathecal IgG synthesis (5.76%)	Normal	Normal	Normal	14	Yes	4	Yes	DIS (MRI), DIT (MRI, CSF)	5.0	16	Fatigue	2 new lesions (5 m after FU)	no new lesions (5 m after FU)	Cladribine 14 m and 2 m prior to FU	—
35	9	22/F	MS	Right optic neuritis	16	2.0	OCB+ M2	Normal	Normal	Abnormal (right)	10–29	No	1–4	—	DIS (MRI), DIT (CSF)	5.0	13	None	No new lesions (3 m after FU)	No new lesions (2 m prior to FU)	Ofatumumab for 12 m	—
38	10	32/F	MS	Left optic neuritis	14	2.0	Intrathecal IgG synthesis (16.78%)	Intrathecal IgM synthesis (0.50%)	Normal	—	10–29	No	1–4	—	DIS (MRI), DIT (CSF)	4.0	12	None	No new lesions (same day as FU)	—	Dimethyl fumarate for 12 m	—
39	11	28/F	MS	Vertigo, painful left eye movement	67	2.0	Intrathecal IgG synthesis (19.02%)	Intrathecal IgM synthesis (0.40%)	Normal	Normal	≥30	No	1–4	no	DIS (MRI), DIT (CSF)	3.0	9	Vertigo, paresthesia in right leg	—	—	Dimethyl fumarate for 6 m	—
42	12	41/M	MS	Left sixth nerve palsy	50	3.0	OCB+ M3	Normal	Normal	—	1–9	No	None	No	DIS (MRI), DIT (CSF)	0.0	8	Persistent mild double vision	No new lesions (same day as FU)	No new lesions (4 m after FU)	Teriflunomide	—
45	13	31/F	MS	Left optic neuritis, transient paresthesia right leg	6	2.0	OCB+ M2, intrathecal IgG synthesis (0.27%)	Intrathecal IgM synthesis (28.90%)	Normal	Abnormal (bilateral)	1–9	No	None	No	DIS (MRI), DIT (CSF)	2.0	11	None	No new lesions (same day as FU)	—	Dimethyl fumarate for 9 m	—
49	14	18/M	MS	Left hemihypoesthesia, hemiataxia, gait disturbance	4	4.5	OCB+ M2, intrathecal IgG synthesis (6.60%)	normal	Normal	Normal	1–9	Yes	1–4	Yes	DIS (MRI), DIT (MRI, CSF)	3.0	8	Persistent left hemihypoesthesia	No new lesions (1 m after FU)	No new lesions (1 m after FU)	Fingolimod for 6 m	—
50	15	31/F	MS	Right optic neuritis	6	2.0	Intrathecal IgG synthesis (21.52%)	Normal	Normal	Abnormal (right)	10–29	No	—	—	DIS (MRI), DIT (CSF)	3.0	8	None	—	—	Dimethyl fumarate for 5 m	—
51	16	40/F	MS	Hypoesthesia in both feet	7	3.0	Intrathecal IgG synthesis (26.31%)	Normal	Normal	Normal	≥30	No	1–4	Yes	DIS (MRI), DIT (MRI, CSF)	1.0	8	Migraine	No new lesions (3 m prior to FU)	No new lesions (1 m prior to FU)	Ocrelizumab 6 m prior to FU	—
53	17	26/F	MS	hypoesthesia face and both arms	19	2.0	Intrathecal IgG synthesis (57.22%)	Normal	Normal	Normal	1–9	Yes	≥5	No	DIS (MRI), DIT (CSF)	1.0	8	Paresthesia in right leg	—	—	Glatiramer acetate for 4 m, then Natalizumab for 2 m	5 additional corticosteroid therapies until 2 m prior to FU
54	18	22/F	MS	Right hemihypoesthesia, hypoesthesia left foot, fine motor disability	22	4.5	OCB+ M2, intrathecal IgG synthesis (34.37%)	Intrathecal IgM synthesis (21.19%)	Normal	Normal	≥30	Yes	>5	Yes	DIS (MRI), DIT (MRI, CSF)	4.0	7	Paresthesia in both hands and feet	No new lesions (same day as FU)	1 new lesion (same day as FU)	Cladribine 5 m prior to FU	—
61	19	23/F	MS	Left hemihypoesthesia	7	2.0	OCB+ M2	Normal	—	—	10–29	Yes	1–4	No	DIS (MRI), DIT (MRI, CSF)	4.0	11	Persistent left hemihypoesthesia	No new lesions (4 m prior to FU)	No new lesions (4 m prior to FU)	Ocrelizumab 6 m prior to FU	—

CE+, contrast-enhancing; CMAP, compound muscle action potential; CSF, cerebrospinal fluid; DIS, dissemination in space; DIT, dissemination in time; DMT, disease-modifying therapy; EDSS, Expanded Disability Status Scale; F, female; Ig, immunoglobulin; M, male; MRC, Medical Research Council muscle scale; M2, pattern 2; M3, pattern 3; OCB+, positive oligoclonal bands; —, not performed.

### MRN analysis

Image analysis was performed blinded to clinical data. The tibial part of the sciatic nerve was manually segmented by a neuroradiologist with 5 years of experience in peripheral nerve imaging. Mean values of signal intensity (SI) were extracted within each region of interest at TE_1_ and TE_2_ and then used to calculate the quantitative parameters of T2 relaxation time (T2app; ms Eq. 1) and proton spin density (PSD; unitless Eq. 2) for each slice according to:


(1) $$T2_{\mathrm{app}}=\frac{{TE}_{2}-{TE}_{1}}{\ln\left(\frac{SI\left({TE}_{1}\right)}{SI\left({TE}_{2}\right)}\right)}$$

(2) $$PSD=\frac{SI\left({TE}_{1}\right)}{\exp\left(\frac{-{TE}_{1}}{T2_{\mathrm{app}}}\right)}$$

### Statistical analysis

Non-parametric Mann–Whitney tests were performed to assess group differences in MRN parameters between patients and healthy controls as well as between patient subgroups. Within-subject differences in patients between the first and second MRN examination were investigated using non-parametric Wilcoxon tests. Spearman’s correlation coefficient was calculated in patients to analyze whether the change in MRN parameters between initial and follow-up scans were associated with the number of T2-lesions on brain or spine MRI, intrathecal IgG rate, the dose of corticosteroids received prior to MRN, the latency of follow-up as well as duration of disease-modifying therapy. Significance was set at *p* ≤ 0.05 except for correlation analyses where the *p*-value was adapted to *p* = 0.01 using Bonferroni’s correction for multiple comparisons. Statistical analyses were performed with Prism (Version 9, GraphPad Software, La Jolla, United States).

## Results

### Study population

Demographic details are outlined in Table 2. Patients and healthy controls did not differ in age (mean: 30.2 ± 7.1 years and 28.4 ± 4.1 years, respectively) or sex (male/female: 16/3 and 16/3) at the time of study enrollment. Patients had sensory and/or motor deficits in at least one leg in 7/19 cases (37%). Mean symptom duration prior to the first MRN study was 16 days (SD 16). The median initial EDSS score was 3.0 (range 2.0–4.5). In CSF studies, intrathecal IgG synthesis was present in 13/19 patients (68%) with a mean rate of 18.2% (SD 19.4). All other patients had evidence of CSF-restricted oligoclonal bands. Intrathecal IgM synthesis was detected in 4/19 patients (21%) with a mean fraction of 2.8% (SD 8.0). NCS was unremarkable in 17/19 patients, and absences of F-waves or A-waves detected in the peroneal nerve in two patients, respectively (Table 1). Intravenous corticosteroid therapy was initiated prior to MRN in 18/19 patients (95%) with a mean duration of 2.9 days (SD 1.6) and a mean dose of 2.8 g (SD 1.5). At follow-up 15/19 (79%) patients received disease-modifying therapy, as outlined in Table 1.

**Table 2:** Demographic, clinical and radiological data.

Characteristic	Patients (*n* = 19)		Healthy controls (*n* = 19)	*p*-value
	At initial diagnosis	At follow-up		
**Demography**				
Age (years)	30.2 ± 7.1	31.3 ± 7.2	28.4 ± 5.1	0.416
Sex (female/male)	16/3		16/3	>0.999
Median time of follow-up (range)	12 (7–16)		n.a.	
**Symptoms (*n*, %)**				
Sensorimotor deficit in one or both legs	7 (37%)	7 (37%)	n.a.	
**Cerebrospinal fluid (*n*, %)**				
Evidence of intrathecal IgG synthesis	13 (68%)	n.a.	n.a.	
Intrathecal IgG rate	18.2 ± 19.4			
Positive oligoclonal bands (patterns 2 or 3)	10 (53%)	n.a.	n.a.	
Evidence of intrathecal IgM synthesis	4 (21%)			
Intrathecal IgM rate	2.8 ± 8.0	n.a.	n.a.	
Evidence of intrathecal IgA synthesis	0 (0%)			
**Nerve conduction study (*n*, %)**				
Abnormal	2 (11%)	n.a.	n.a.	
Normal	16 (84%)	n.a.	n.a.	
Not performed	1 (5%)	n.a.	n.a.	
**Steroid therapy prior to MRN (*n*, %)**	18 (95%)		n.a.	
Dose (g)	2.8 ± 1.5	n.a.	n.a.	
Duration (days)	2.9 ± 1.6	n.a.	n.a.	
**Brain MRI**				
Number of T2w-lesions	20 ± 14	n.a.	n.a.	
Presence of contrast-enhancing lesions	8 (42%)	n.a.	n.a.	
**Spine MRI**				
Number of T2w-lesions	2 ± 2	n.a.	n.a.	
Presence of contrast-enhancing lesions	6 (32%)	n.a.	n.a.	
Not performed	3 (16%)	n.a.	n.a.	

Values are mean ± standard deviation. Ig, immunoglobulin; MRN, magnetic resonance neurography; T2w, T2-weighted.

### Brain and spine MRI

Median latency from first MRN to brain MRI was 4 days (range 0–24) and to spine MRI 2 days (range 0–6). All patients had at least two T2/FLAIR lesions in MS-typical locations on brain MRI (mean 20, SD 14), of which 8 studies showed contrast-enhancing lesions (42%). On spine MRI, 11 patients had MS-typical medullary T2 lesions (mean 2, SD 2) with contrast-enhancement in 6 cases (32%). Follow-up brain MRI was available in 15 patients of whom 12 had no new T2/FLAIR lesions, while spine MRI was available in 11 patients without evidence of new lesions in 9 cases (details in Table 1).

### MRN in patients and healthy volunteers

**Figure 1:**
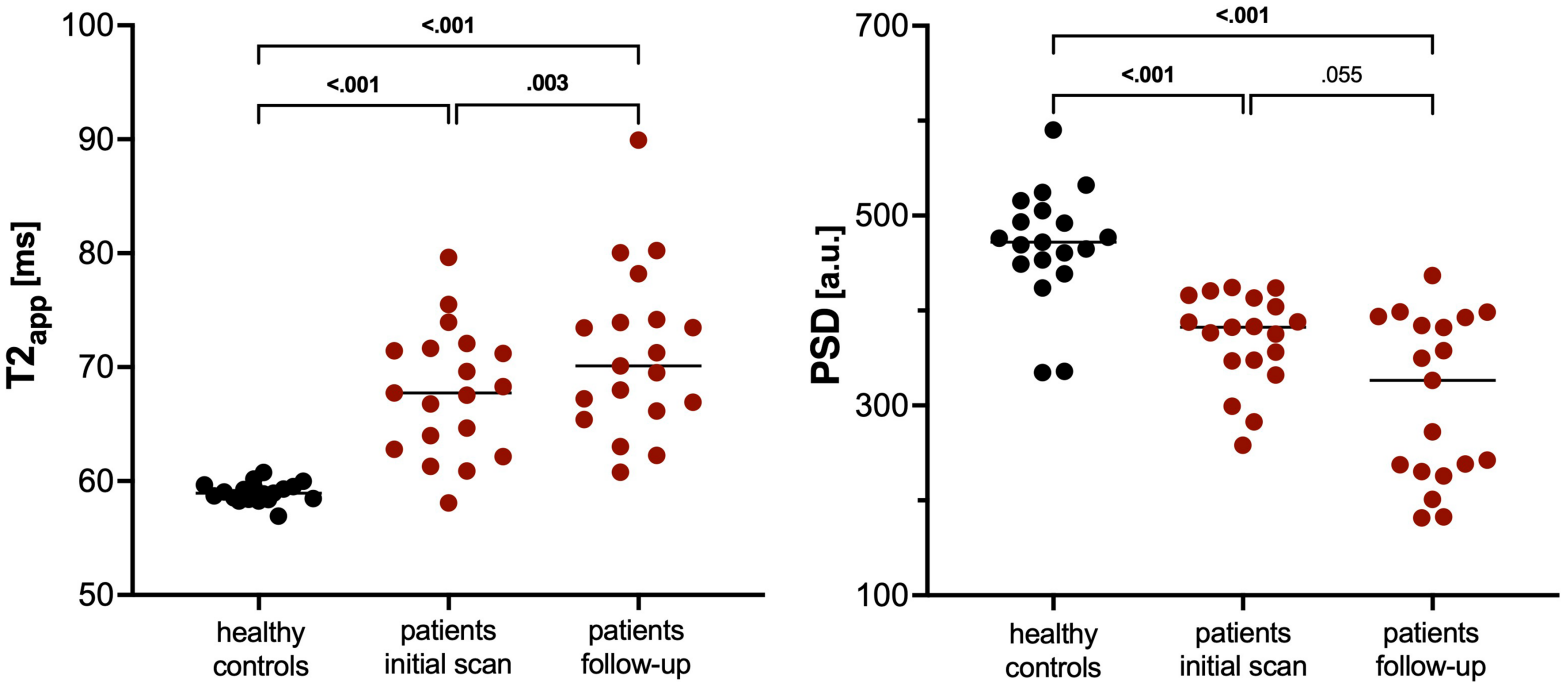
Scatter dot plots of T2 relaxation time (T2app) and proton spin density (PSD) in healthy volunteers and patients with multiple sclerosis at initial scan as well as at follow-up. Lines indicate the median. *p*-values were obtained using Kruskal–Wallis tests between patients and controls or using Wilcoxon tests between patient groups.

**Figure 2:**
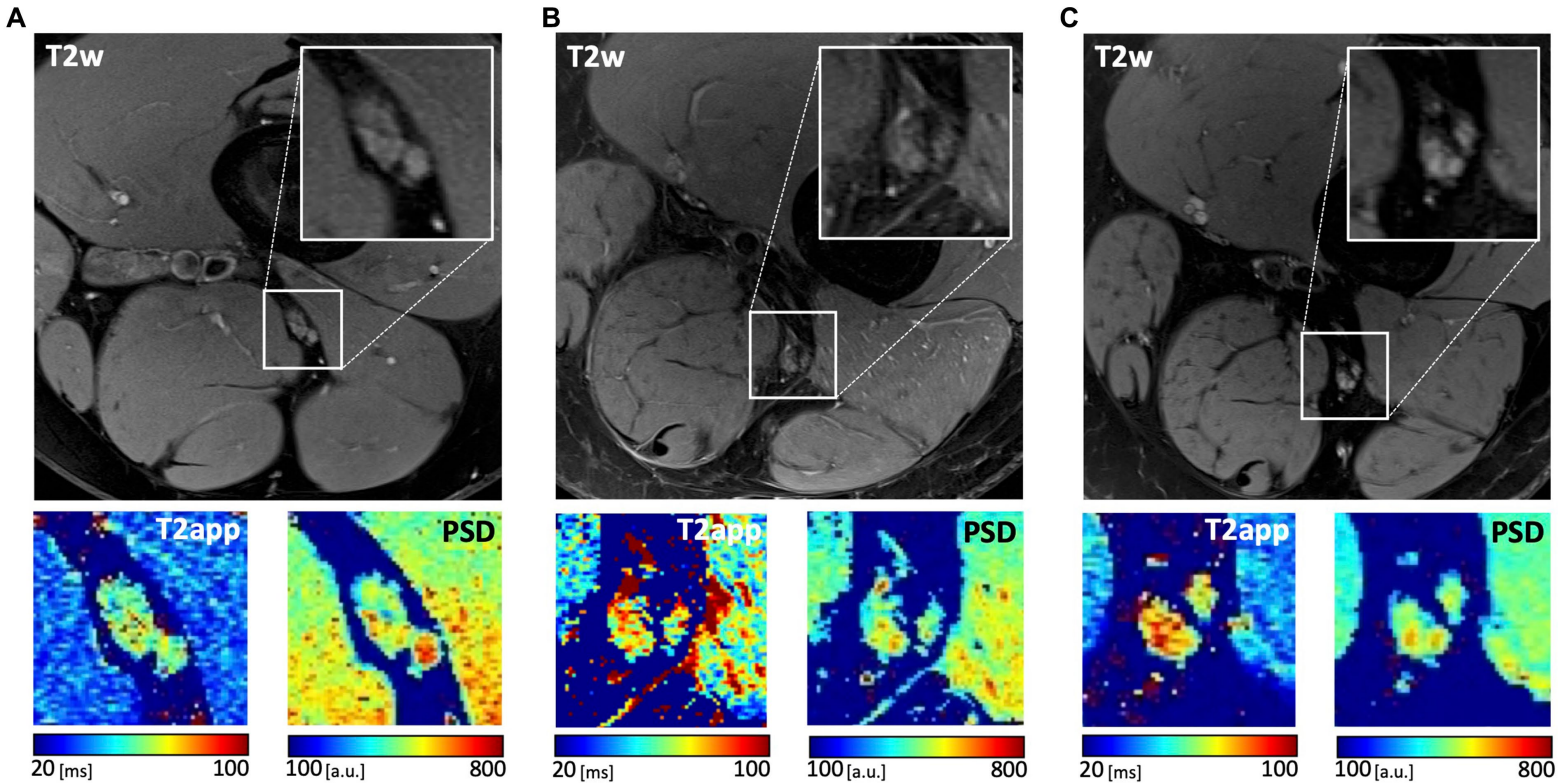
Representative images of the sciatic nerve at distal thigh level in a healthy participant **(A)** and in patient #14 at initial diagnosis **(B)** and at follow-up **(C)**. Axial fat-saturated T2-weighted (T2w) images of the nerve as well as parameter maps of T2app and PSD with corresponding calibration bars are shown.

**Figure 3:**
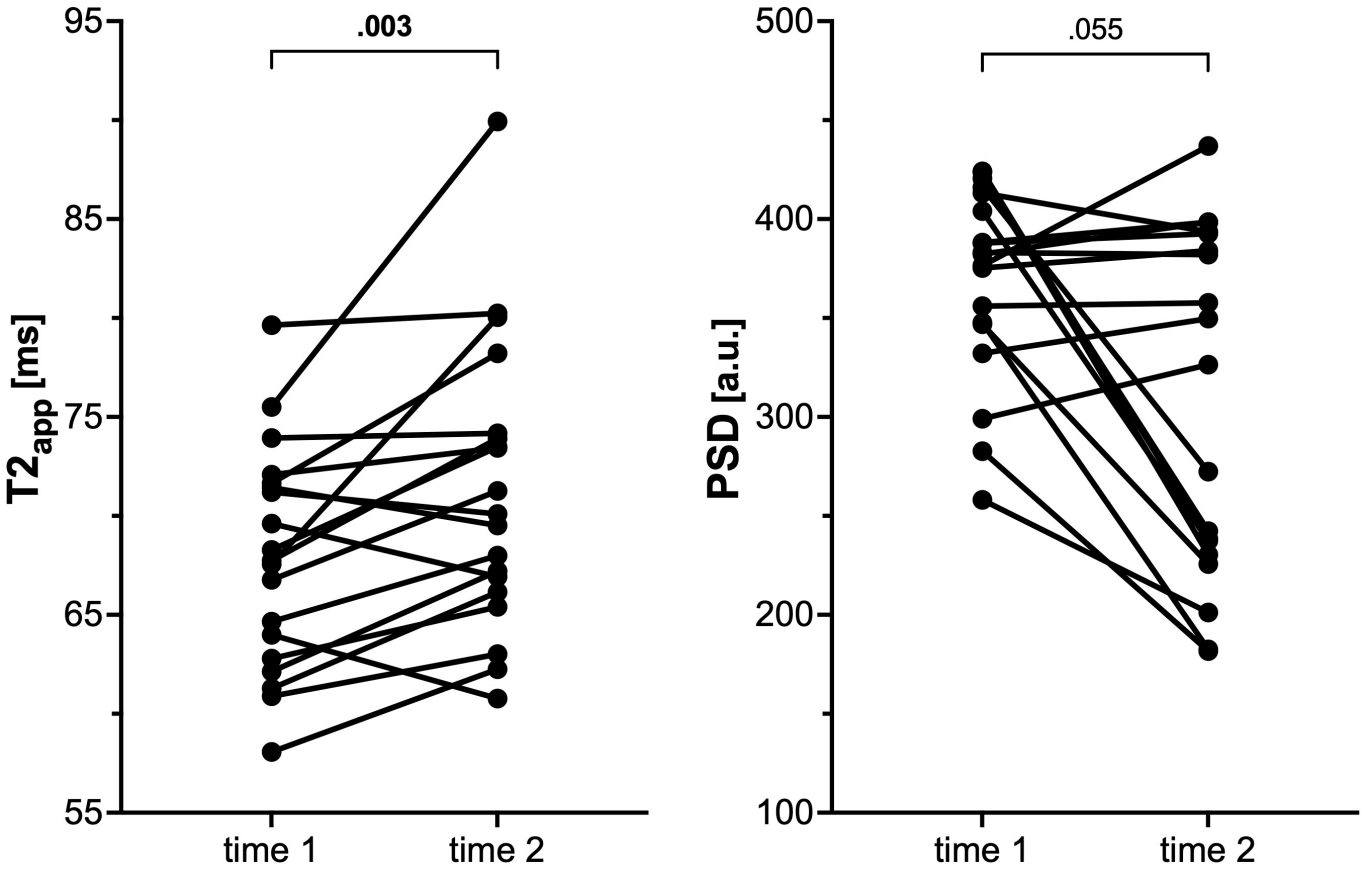
Graph showing the change in T2 relaxation time (T2app) and proton spin density (PSD) within individual patients from initial scan (time 1) to follow-up (time 2). *p*-values were obtained using Wilcoxon tests.

Patients showed increased T2app compared to healthy controls at initial diagnosis and at follow-up (mean 67.9 ms ±5.6 and 71.3 ms ±7.3 vs. 59.0 ± 0.9, *p* < 0.001; Table 3 and Figure 1). In contrast, PSD was decreased in patients at both time points in comparison to healthy volunteers (mean 369.3 ± 48.5 and 306.9 ± 86.5 vs. 468.9 ± 60.5, *p* < 0.001). Representative examples are depicted in Figure 2. Compared to the initial scan, T2app further increased in patients at follow-up (mean difference 3.4 ms, *p* = 0.003; Figure 3). PSD further declined by at least 10% in a subgroup of patients (9/19, 47%), remained stable (i.e., within 10% range) in another 9/19 patients, while it increased in a single patient by 16%. Subgroup analyses of patients did not reveal any significant differences nor correlations between MRN parameters and clinical or radiographic features (Table 4).

**Table 3:** Results of Kruskal–Wallis test between patients and healthy subjects.

MRN parameter	Healthy controls	Patients		*p*-value^*^
		At initial diagnosis	At follow up	
**T2 relaxation time (ms)**				
	59.0 ± 0.86	67.9 ± 5.6	71.3 ± 7.3	**<0.001**
**Proton spin density (a.u.)**				
	468.9 ± 60.5	369.3 ± 48.5	306.9 ± 86.5	**<0.001**

Values are mean ± SD. Bold figures indicate significance.

**Table 4:** Results of correlation analyses between relative change in T2 relaxation time or PSD and clinical/CNS imaging parameters in patients using Spearman’s correlation coefficient.

		T2 relaxation time	Proton spin density
Brain MRI lesions	*r*	0.04	0.00
	*p*	0.88	0.99
Spine MRI lesions	*r*	−0.29	−0.46
	*p*	0.30	0.09
Intrathecal IgG synthesis [%]	*r*	0.15	0.02
	*p*	0.53	0.95
Dose of corticosteroids [g]	*r*	0.04	−0.21
	*p*	0.88	0.39
Time to follow-up [months]	*r*	0.02	0.52
	*p*	0.94	0.02

The threshold of significance was adapted to *p* ≤ 0.01 using Bonferroni correction for multiple comparisons. DMT, disease-modifying therapy.

## Discussion

Multiple sclerosis is characterized by demyelinating lesions of the CNS. Recently, increasing evidence points towards an additional involvement of peripheral nerves in patients with MS (2–10). In line with previous cross-sectional imaging studies, this first longitudinal investigation provides novel insights into the evolution of PNS changes using quantitative T2 relaxometry. At 1 year follow up, both MRN biomarkers of T2app and PSD indicate ongoing affection of peripheral nerves with individual pace of severity. Lack of association with CNS demyelination or disease-modifying therapy strengthens the hypothesis of an inflammatory etiology of the detected PNS changes.

As shown by three recent imaging studies using quantitative MR-neurography, peripheral nerve changes can be detected in early and chronic stages of MS (2–4). The current investigation confirms these previous findings and complements them by a longitudinal perspective from initial diagnosis up to a median of 1 year follow-up. While the T2 relaxation time showed a rather consistent longitudinal increase in the study population, the other analyzed MRN biomarker of PSD was dominated by two distinct subgroups with either ongoing or stable changes. However, we could not clearly identify any separating factor among the included clinical/neurological parameters, CSF markers, electrophysiological studies, CNS lesion load nor therapy regimen. Yet, some analyses were limited by the small sample size of the sub-populations. Future studies should therefore further explore which factors best predict the appearance and individual course of PNS involvement in order to develop even more tailored treatment regimens of MS patients.

Subtyping of MS requires objective biomarkers with high diagnostic accuracy. Quantitative MRN sequences such as T2-relaxometry could provide important novel biomarkers to detect and monitor peripheral nerve affection in MS. It proved to be highly sensitive in various peripheral neuropathies (11, 12, 14, 22) as well as methodologically reliable and robust (23, 24). T2-relaxometry is a quantitative measure of water content and thereby provides biomarkers of nerve tissue integrity (22, 25, 26). The specific pattern of the derived biomarkers T2app and PSD hints at the underlying pathomechanism, e.g., increased T2app was found in peripheral nerve swelling due to amyloidosis or multifocal motor neuropathy (18, 27, 28) and decreased T2app and PSD was detected in predominant axonal damage due to neurodegenerative disorders (14, 22). In line with our findings, acute CNS lesions in MS patients are characterized by an increase in T2app reflecting the demyelinating process (29–31). This pattern remained at 1 year follow-up in our cohort, but may invert over a longer period of time, as observed by a previous MRN study in chronic MS patients with mean 6.8 years of disease history (2). Such a temporal evolution of T2 relaxometry parameters was also described in central MS lesions (31–33). MRN may thus become a valuable diagnostic tool to detect and monitor PNS affection in MS patients, helping to prevent further, possibly irreversible peripheral nerve damage.

Limitations of this study are the moderate sample size precluding detailed subgroup analyses, particularly regarding factors that predict the evolution of T2app and PSD changes over time. Especially, the influence of different therapy regimens should be further elucidated in subsequent studies as well as the possible influence of corticosteroid treatment in the acute setting. Follow-up brain and spine imaging was not available at the same day as MRN in all cases, which would have improved the correlation between PNS and CNS lesions. The dual-echo relaxometry sequence and mono-exponential fit may lead to minor inaccuracies due to B1 field inhomogeneities (34, 35). Absolute T2-relaxometry values, primarily PSD, cannot be easily generalized as they depend on scanner hardware and sequence parameters. Finally, additional MRN sequences such as diffusion-weighted imaging could complement the perspective on pathophysiological changes in peripheral nerves of MS patients.

In conclusion, this study confirms the occurrence of additional peripheral nerve involvement in patients with early-stage MS. Longitudinal data collected over 1 year of follow-up point at sub-populations more susceptible to PNS affection. The pattern of quantitative MRN parameters suggests predominant demyelination and strengthens the hypothesis of an inflammatory co-affection or rather co-demyelination of the PNS. Quantitative MRN sequences could provide non-invasive biomarkers to detect and monitor peripheral nerve involvement in MS.

## Data availability statement

The raw data supporting the conclusions of this article will be made available by the authors, without undue reservation.

## Ethics statement

The studies involving humans were approved by the Ethical Board of the University Hospital Heidelberg (S-405/2012). The studies were conducted in accordance with the local legislation and institutional requirements. The participants provided their written informed consent to participate in this study.

## Author contributions

OF: Data curation, Formal analysis, Funding acquisition, Investigation, Project administration, Validation, Visualization, Writing – original draft, Writing – review & editing. JH: Conceptualization, Funding acquisition, Methodology, Project administration, Resources, Supervision, Validation, Writing – review & editing. MW: Investigation, Project administration, Resources, Supervision, Validation, Writing – review & editing. GS: Data curation, Investigation, Project administration, Resources, Validation, Writing – review & editing. BW: Resources, Supervision, Validation, Writing – review & editing. WW: Funding acquisition, Resources, Supervision, Validation, Writing – review & editing. MB: Conceptualization, Funding acquisition, Methodology, Resources, Software, Supervision, Validation, Writing – review & editing. SH: Conceptualization, Funding acquisition, Methodology, Resources, Software, Supervision, Validation, Visualization, Writing – review & editing. LJ: Conceptualization, Formal analysis, Investigation, Methodology, Project administration, Resources, Validation, Writing – original draft, Writing – review & editing.
