# Supplementary figures and images for: Evolution of peripheral nerve changes in early multiple sclerosis—a longitudinal MR neurography study
Source: Front Neurol. 2024 May 3;15:1335408. doi: 10.3389/fneur.2024.1335408 (PMC11100769; doi:10.3389/fneur.2024.1335408)

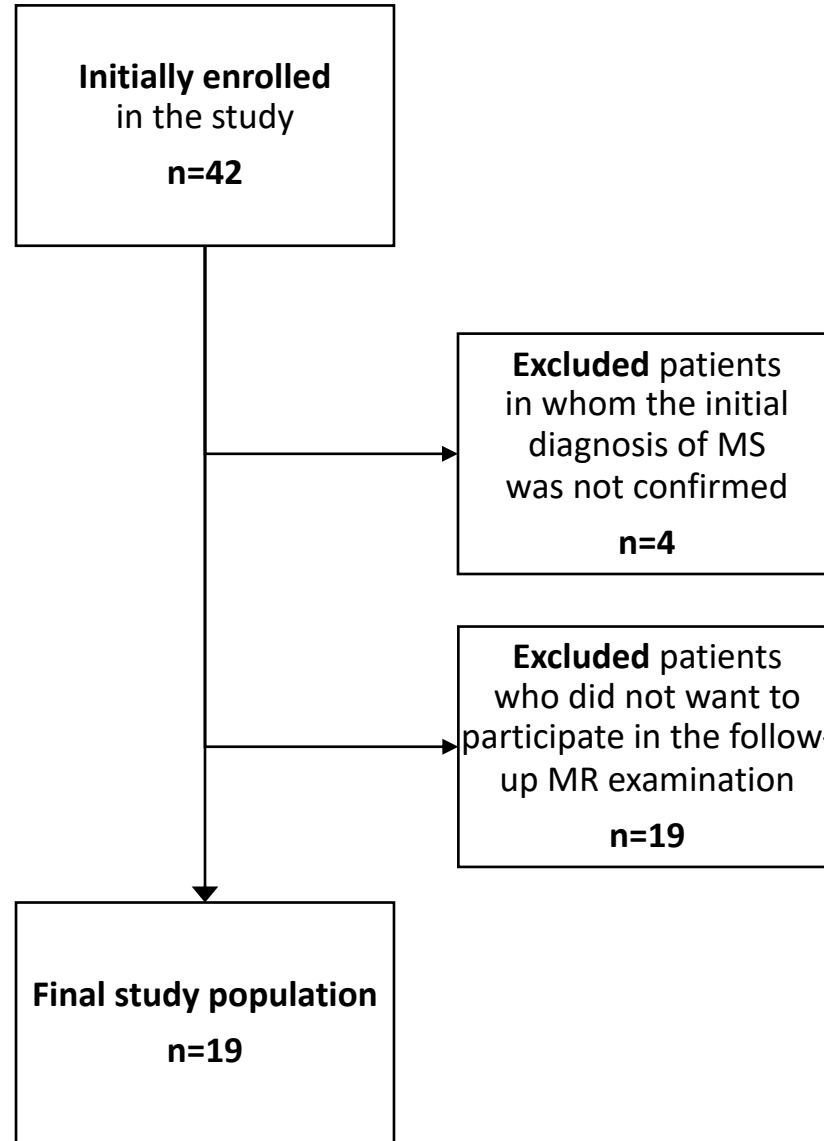

Supplement: Supplementary file 1 [file Presentation_1.pdf]
